# Ibuprofen disrupts a WNK1/GSK3β/SRPK1 protein complex required for expression of tumor-related splicing variant RAC1B in colorectal cells

**DOI:** 10.18632/oncotarget.27816

**Published:** 2020-11-24

**Authors:** Vânia Gonçalves, Andreia F.A. Henriques, Paulo Matos, Peter Jordan

**Affiliations:** ^1^Department of Human Genetics, National Health Institute Dr. Ricardo Jorge, Lisbon, Portugal; ^2^BioISI-Biosystems & Integrative Sciences Institute, Faculty of Sciences, University of Lisbon, Lisbon, Portugal; ^*^These authors contributed equally to this work

**Keywords:** ibuprofen, protein kinase, RAC1B, alternative splicing, colorectal cancer cells

## Abstract

A major risk factor promoting tumor development is chronic inflammation and the use of nonsteroidal anti-inflammatory drugs (NSAID), including ibuprofen, can decrease the risk of developing various types of cancer, including colorectal cancer (CRC). Although the molecular mechanism behind the antitumor properties of NSAIDs has been largely attributed to inhibition of cyclooxygenases (COXs), several studies have shown that the chemopreventive properties of ibuprofen also involve multiple COX-independent effects. One example is its ability to inhibit the alternative splicing event generating RAC1B, which is overexpressed in a specific subset of BRAF-mutated colorectal tumors and sustains cell survival. Here we describe the mechanism by which ibuprofen prevents RAC1B alternative splicing in a BRAF mutant CRC cell line: it leads to decreased translocation of SRPK1 and SRSF1 to the nucleus and is regulated by a WNK1/GSK3β/SRPK1 protein kinase complex. Surprisingly, we demonstrate that ibuprofen does not inhibit the activity of any of the involved kinases but rather promotes disassembly of this regulatory complex, exposing GSK3β serine 9 to inhibitory phosphorylation, namely by AKT, which results in nuclear exclusion of SRPK1 and SRSF1 hypophosphorylation. The data shed new light on the biochemical mechanisms behind ibuprofen’s action on alternative spliced RAC1B and may support its use in personalized approaches to CRC therapy or chemoprevention regimens.

## INTRODUCTION

Cancer is the second leading cause of death globally [1] and one major risk factor for tumor development is chronic inflammation [2]. This is particularly true for malignancies of the digestive system, including liver and the gastrointestinal tract. For example, patients with inflammatory bowel disease have a 2 to 8 times higher risk of developing colorectal cancer (CRC), compared to the general population [3]. A long term use of nonsteroidal anti-inflammatory drugs (NSAIDs), like ibuprofen and aspirin, which are among the most commonly prescribed medications worldwide, was shown to provide chemoprevention against various types of cancer [4]. In particular, several population-based studies have shown that regular, long-term users of NSAIDs have a significantly lower risk of developing colorectal adenomatous polyps and CRC than non-users [5–7]. The molecular mechanism behind the antitumor properties of NSAIDs has been largely attributed to the inhibition of prostaglandin synthesis by blocking the enzymes cyclooxygenases (COXs), which are involved in the inflammatory response [8]. COX-1 isoform is constitutively expressed in most tissues and the prostaglandins it produces have physiological roles in the maintenance of the gastric mucosa, blood vessel regulation and kidney function [9]. Another isoform, COX-2, is induced during the inflammatory response but more persistently expressed under pathological conditions including chronic inflammation and cancer [10]. Ibuprofen, like most NSAIDs, inhibits both COX isoforms so that side-effects such as intestinal bleeding or cardiovascular disease can occur, questioning the long-term use of NSAIDs for cancer chemoprevention [8]. Interestingly, some NSAIDs were reported to inhibit tumor growth by targeting other cellular processes and elucidation of the underlying biochemical processes could lead to the development of safer and more efficacious drugs for cancer chemoprevention or adjuvant therapy [11]. In case of ibuprofen, numerous studies have shown that its cancer chemopreventive properties are much more complex and involve multiple COX-independent effects [8]. These can act through various cell cycle- and apoptosis-regulating pathways, including β-catenin, NF-κB, and p53, leading to changes in gene expression [12]. Previously, our team showed that another COX-independent effect of ibuprofen is its ability to inhibit the alternative splicing event generating RAC1B, a tumor-associated variant of the signaling RAC1 GTPase [12, 13]. RAC1B results from the in-frame inclusion of an alternative exon 3b, which produces a highly activated variant that is overexpressed in a specific subset of colorectal tumors, characterized by a serrated polyp morphology and the presence of activating mutations in BRAF [14, 15]. Even small changes in RAC1B protein levels will generate a predominantly GTP-loaded and signaling competent conformation that preferentially stimulates the transcription factor NF-κB and is required to sustain cell survival in BRAF mutant CRC cells [16].

Here we unveiled the molecular mechanisms of how ibuprofen interferes with alternative splicing in BRAF mutant CRC cells. We show that ibuprofen disrupts a signal transduction pathway by, unexpectedly, interfering with the assembly of a protein kinase complex, composed by WNK1, GSK3β and SRPK1. This leads to changes in the subcellular localization of splicing factor SRSF1, which promotes inclusion of exon 3b into the mRNA and subsequent expression of RAC1B.

## RESULTS

### Ibuprofen inhibits RAC1B overexpression by SRSF1-mediated regulation of alternative splicing

**Figure 1:**
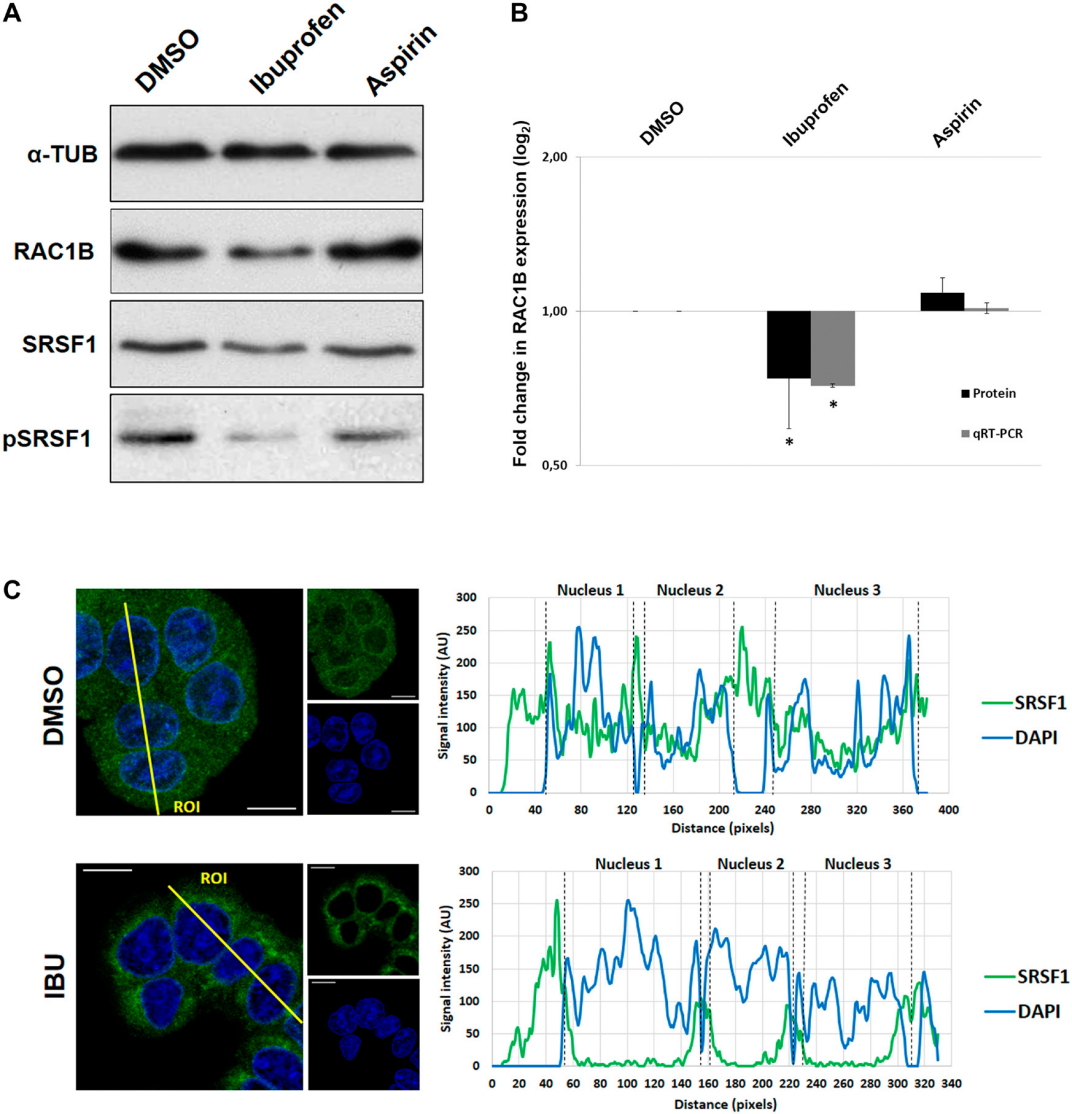
Effect of Ibuprofen treatment on RAC1B and SRSF1. The human colorectal adenocarcinoma cell line HT29 was incubated for 48 h with a DMSO control solvent, 500 μM ibuprofen, or 500 μM aspirin. (**A**) Western blot detection of endogenous levels of RAC1B and SRSF1 proteins together with α-tubulin as loading control. Note the reduction of both RAC1B and pSRSF1 in ibuprofen-treated lane. (**B**) Graphical display of the Western blot analysis of RAC1B protein band intensities, quantified and normalized to the DMSO control, along with qRT-PCR detection of the ratio between endogenous RAC1B and total RAC1 transcript levels. Note the decrease of both RAC1B protein and transcript in ibuprofen-treated condition. All shown data represent means ± SEM: ^*^ *P* < 0.05. (**C**) Subcellular localization of endogenous SRSF1 protein. Shown is the colored overlay of two confocal immunofluorescence images (left), which detected cell nuclei in blue (DAPI) and the localization of endogenous SRSF1 protein in green (anti-SF2/ASF from Santa Cruz Biotechnology). The nucleus and cytoplasm distribution of the two fluorescent signals was analyzed along optical sections (yellow lines) across several cells by plotting pixel intensities along the traced path (right graphs). In control cells, SRSF1 signals (green) were localized to both the cytosol and the cell nucleus (blue); however, in ibuprofen-treated cells, nuclear signals for SRSF1 are nearly absent (AU: arbitrary units; DAPI: 4',6-diamidino-2-phenylindole; DMSO: dimethyl sulfoxide; IBU: ibuprofen).

Previously, we described that the anti-inflammatory drug ibuprofen inhibited RAC1B expression in HT29 colorectal tumor cells [13], but the mechanistic details of this inhibition remained to be determined. First, we confirmed that ibuprofen affected RAC1B protein and transcript levels, as expected from an alternative splicing event. In Figure 1A, we observe the decrease in RAC1B protein levels following ibuprofen treatment and in Figure 1B, we confirmed a proportional reduction in the ratio of RAC1B to total RAC1 mRNA levels for that same condition. Aspirin was used as control because it is also a non-steroidal anti-inflammatory drug but described as having no effect in RAC1B expression [13]. Our previous data also recognized SRSF1 as a prime regulator of RAC1B expression in colorectal cells, and that its phosphorylation and subsequent nuclear translocation were required to promote the inclusion of alternative exon 3b into RAC1 pre-mRNA [17]. Consistently, here we observed that in ibuprofen-treated HT29 cells the nuclear localization of SRSF1 was dramatically reduced. (Figure 1C). SRSF1 has been predominantly described as a nuclear phospho-protein and we previously described loss of nuclear localization of transfected epitope tagged SRSF1 upon depletion of the phosphorylating kinase SRPK1 [17]. This decrease was clearly observed with the new antibody capable of detecting endogenous SRSF1. This nuclear absence of SRSF1 was concomitant with an ibuprofen-induced decrease in its phosphorylation and overall steady state abundance, which were not observed upon a similar treatment with aspirin (Figure 1A). These results suggest that ibuprofen inhibits RAC1B splicing through alterations in the phosphorylation state of SRSF1 and in its subcellular localization.

### Ibuprofen prevents RAC1B splicing by decreasing SRPK1 nuclear translocation

**Figure 2:**
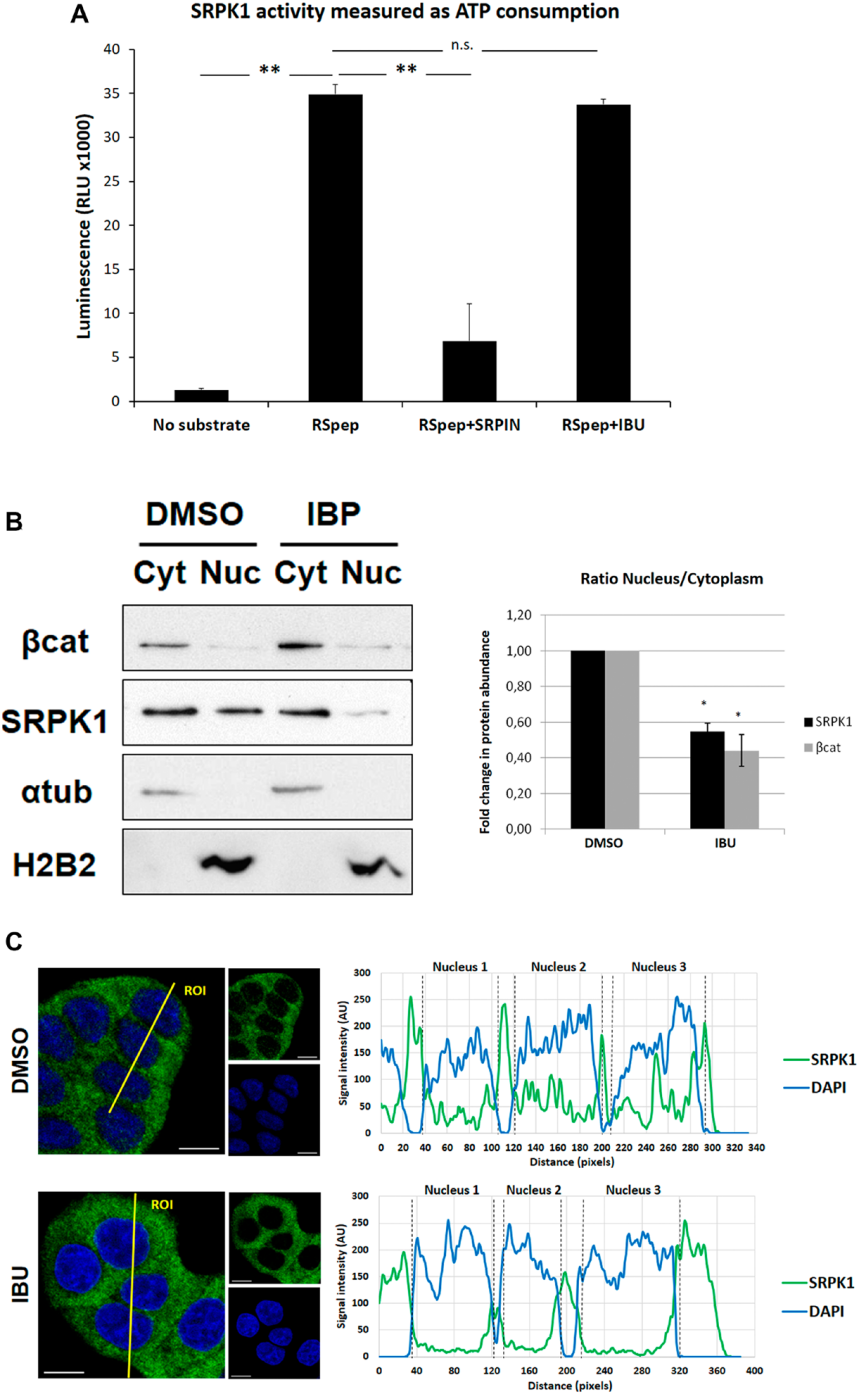
Effect of ibuprofen treatment on SRPK1 catalytic activity and subcellular localization. (**A**) The ADP-Glo™ Kinase Assay kit (Promega) was used to measure the influence of ibuprofen treatment on the catalytic activity of the kinase SRPK1, as described in the experimental procedures. A known SRPK1 synthetic substrate RS Repeat peptide (Abcam) and the specific SRPK inhibitor SRPIN340 (kindly provided by Dr. Masatoshi Hagiwara) were used as controls. Shown is a graphical display of the luminescence measured, representing the ATP consumption during the kinase reaction. Note that there was no significant difference between the ATP consumption for the kinase reaction with ibuprofen (RSpep+IBU bar) when compared to the control with DMSO (RSpep bar), while the treatment with the specific inhibitor of SRPK kinases produced very significant decrease (RSpep+SRPIN bar). All shown data represent means ± SEM from three independent experiments: ^**^ *P* < 0.01. (**B**) HT29 cells were incubated for 48 h with either a DMSO control solvent or 500 μM ibuprofen and cellular fractionation assays were performed to assess endogenous SRPK1 protein levels in the nucleus vs the cytoplasm using β-catenin as positive control. The upper panel shows a Western blot detection of endogenous levels of SRPK1 and β-catenin proteins together with α-tubulin and Histone 2B (H2B) as controls for constitutive cytoplasmic and nuclear proteins, respectively. The lower panel shows a graphical display of the ratio between the nucleus and the cytoplasmic fractions for both proteins, obtained by densitometry of Western blots bands. All shown data represent means ± SEM: ^*^ *P* < 0.05. (**C**) HT29 cells were incubated for 48 hours with either a DMSO control solvent or 500 μM ibuprofen and confocal immunofluorescence microscopy analysis was performed. Shown are the colored overlay and corresponding two images (left), which detected cell nuclei in blue (DAPI) and the localization of endogenous SRPK1 protein in green (anti-SRPK1 from BD Transduction Laboratories). The nucleus and cytoplasm distribution of the two fluorescent signals was analyzed along optical sections (yellow lines) across several cells by plotting pixel intensities along the traced path (right graphs). In control cells, SRPK1 signals (green) were localized to both the cytosol and the cell nucleus (blue); however, in ibuprofen-treated cells, nuclear signals for SRPK1 are nearly absent (AU: arbitrary units; Cyt: Cytoplasm fraction; DAPI: 4',6-diamidino-2-phenylindole; DMSO: dimethyl sulfoxide; IBU: ibuprofen; Nuc: Nuclear fraction; RLU: relative light units; RSpep: RS repeat synthetic peptide; SRPIN: SRPK inhibitor SRPIN340).

SRPK1 is a well-known protein kinase that phosphorylates several SR proteins, including SRSF1 [18]. Moreover, we previously demonstrated that phosphorylation of SRSF1 by SRPK1 is required for its nuclear translocation and induction of RAC1B splicing [17]. We therefore asked whether ibuprofen treatment could be directly inhibiting SRPK1 kinase activity. To test this under controlled conditions, we used an *in vitro* kinase assay to measure the activity of recombinant SRPK1 towards the commonly used synthetic RSpeptide (Abcam) substrate. We observed that, in contrast to the highly selective, ATP-competitive SRPK inhibitor SRPIN340 [19], the presence of ibuprofen did not interfere with SRPK1 activity (Figure 2A).

Notwithstanding, a previous report has revealed that SRPK1 activity towards SRSF1 requires its own shuttling to the nucleus from the cytoplasm [20]. We thus asked whether it could be the nuclear translocation of SRPK1 that was being affected by ibuprofen treatment, preventing it from sustaining SRSF1 phosphorylation in the nucleus, which might be required for nuclear retention of SRSF1 and subsequent promotion of RAC1B alternative splicing.

To test this we isolated the nuclear and cytoplasmic cellular fractions and analyzed the levels of SRPK1 in both compartments, comparing vehicle- and ibuprofen-treated HT29 cells. Although the majority of SRPK1 was detected in the cytoplasm in untreated cells, a fraction accumulated in the nucleus and this fraction was excluded upon ibuprofen treatment, leading to its complete accumulation in the cytoplasm (Figure 2B). Consistent with our hypothesis, we observed a roughly 2-fold decrease in the nucleus/cytoplasm ratio of SRPK1 levels in ibuprofen treated cells (Figure 2B). Notably, a similar decrease occurred on the ratio of nuclear versus cytosolic β-catenin levels in these cells. Although we cannot exclude that ibuprofen promoted stabilization of cytosolic β-catenin, our results are compatible with a previous report [21] showing that nuclear translocation of β-catenin was prevented by ibuprofen treatment. In contrast, the localization of two typical nuclear or cytosolic marker proteins, histone 2B or α-tubulin, respectively, was not affected in ibuprofen-treated cells.

Confocal microscopy confirmed that, similar to what was observed for SRSF1, ibuprofen treatment prevented nuclear translocation of SRPK1 in HT29 cells (Figure 2C).

### Ibuprofen prevents RAC1B expression through indirect inhibition of GSK3β

**Figure 3:**
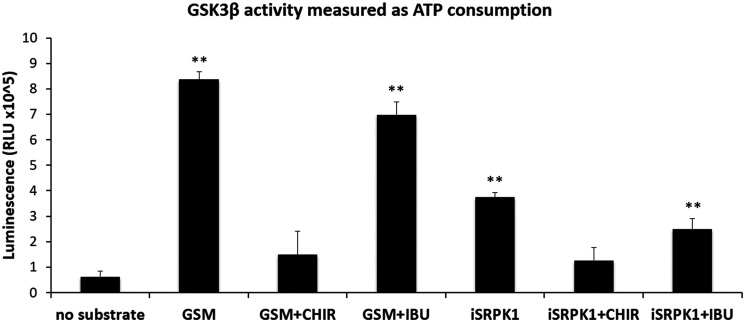
Effect of ibuprofen treatment on GSK3β catalytic activity. Kinase-Glo^®^ Luminescent Kinase Assay (Promega) was used to measure the influence of ibuprofen treatment on the catalytic activity of the kinase GSK3β as described in the experimental procedures. A known GSK3 synthetic substrate peptide GSM (Millipore) and the specific GSK3 inhibitor CHIR99021 (BioVision) were used as controls. The ability of GSK3β to phosphorylate SRPK1 was tested in the presence of the specific SRPK inhibitor SRPIN340 in order to abolish SRPK1 kinase activity (iSRPK1) and allowing the specific measurement of GSK3β kinase activity. Shown is a graphical display of the luminescence measured from three independent experiments, representing the ATP consumption during the kinase reaction. Note that there was a significant increase in the ATP consumption when GSK3β was incubated with substrate, either GSM or iSRPK1, both in the presence of the control DMSO (GSM and iSRPK1 bars) or of ibuprofen (GSM+IBU and iSRPK1+IBU bars). In contrast, in the presence of the specific inhibitor of GSK3β kinase almost no ATP was consumed in the assays with either substrate (GSM+CHIR and iSRPK1+CHIR bars). All shown data represent means ± SEM: ^**^ *P* < 0.01 (DMSO: dimethyl sulfoxide; CHIR: CHIR99021 (GSK3 inhibitor); GSM: GSK3 substrate peptide; IBU: ibuprofen; iSRPK1: SRPK1 + SRPIN340; RLU: Relative Light Units).

Recently, we found that besides SRPK1, the depletion of GSK3β also decreased RAC1B alternative splicing through changes in splicing factor SRSF1, and speculated that GSK3β could act upstream of SRPK1 in the regulation of RAC1B alternative splicing [17]. To clarify this aspect, we tested *in vitro* whether GSK3β could directly phosphorylate SRPK1. Using the Kinase-Glo^®^ Luminescent Kinase Assay (Promega) that measures how much ATP remains after the kinase reaction, we found that incubation of GSK3β with GSM, a synthetic glycogen synthase-derived GSK3 substrate peptide, clearly increased ATP consumption (Figure 3). This activity was also observed when SRPIN340-inactivated SRPK1 (iSRPK1) was included as the substrate (Figure 3). Moreover, the activity towards both GSM and iSRPK1 was suppressed by co-incubation with the GSK3-specific inhibitor CHIR99021 (Figure 3). These results show that GSK3β can directly phosphorylate SRPK1.

We next asked whether ibuprofen could directly inhibit GSK3β kinase activity towards these substrates. However, we found that, in contrast to the CHIR99021 inhibitor, the presence of ibuprofen did not significantly inhibit GSK3β ability to phosphorylate either GSM or iSRPK1 (Figure 3).

**Figure 4:**
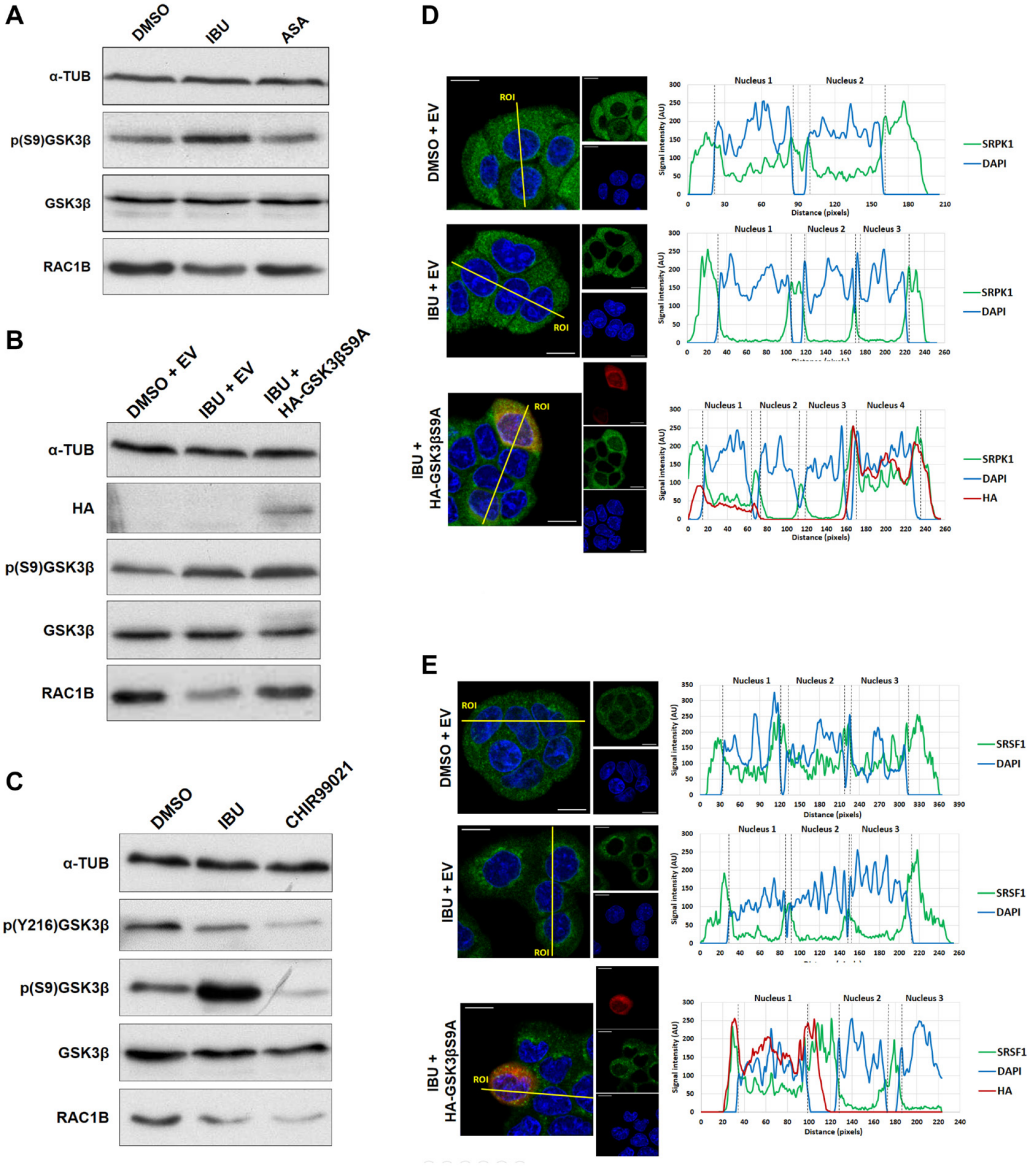
Ibuprofen requires GSK3β inhibition for decreasing RAC1B levels. (**A**) HT29 cells were incubated for 48 h with either a DMSO control solvent, 500 μM ibuprofen or 500 μM aspirin and analyzed by Western blot for their effect on endogenous GSK3β, p (S9) GSK3β, RAC1B, SRSF1 and pSRSF1 protein levels together with α-tubulin as loading control. (**B**) HT29 cells were incubated with either a DMSO control solvent or 500 μM ibuprofen and after 24 h transfected with HA-tagged GSK3βS9A or a control empty vector. Another 24 h later, cells were analyzed by Western blot to assess the endogenous levels of RAC1B and GSK3β protein as well as of GSK3β S9 phosphorylation. Overexpression of GSK3βS9A alone had no effect on RAC1B levels. (**C**) HT29 cells were treated with ibuprofen (500 μM) or CHIR99021 (10 μM) for 48 h and analyzed by Western blot for their effect on endogenous RAC1B, GSK3β, p (S9) GSK3β and p (Y216) GSK3β protein levels together with α-tubulin as loading control. Note that CHIR99021 inhibited GSK3β activity by preventing autophosphorylation at Y216, independent of S9 phosphorylation, and this was sufficient to decrease RAC1B levels. (**D** and **E**) HT29 cells were incubated with either a DMSO control solvent or 500 μM ibuprofen and 24 h later transfected with HA-tagged GSK3βS9A or a control empty vector (the same as B). Another 24 h later, cells were analyzed by confocal immunofluorescence microscopy. Shown are the colored overlay and corresponding confocal immunofluorescence images (left), which display cell nuclei in blue (DAPI), the localization of (D) endogenous SRPK1 protein in green (anti-SRPK1 from BD Transduction Laboratories), or (E) endogenous SRSF1 protein in green (anti-SF2/ASF from Santa Cruz Biotechnology), and the transfected HA-tagged GSK3βS9A in red (anti-HA from Sigma-Aldrich). The nucleus and cytoplasm distribution of the three fluorescent signals was analyzed along optical sections (yellow lines) across several cells by plotting pixel intensities along the traced path (right graphs). In control and transfected HA-GSK3βS9A cells, SRPK1 and SRSF1 signals (green) were localized to both the cytosol and the cell nucleus (blue); however, in ibuprofen-treated cells, nuclear signals for SRPK1 and SRSF1 are nearly absent (ASA: aspirin; AU: arbitrary units; DAPI: 4',6-diamidino-2-phenylindole; DMSO: dimethyl sulfoxide; EV: empty vector; IBU: ibuprofen).

Based on these data we considered other modes of action of ibuprofen. Interestingly, it had previously been reported that ibuprofen treatment of SW480 colorectal tumor cells produced an increase in the inhibitory phosphorylation of GSK3β at serine 9 [21]. We therefore evaluated GSK3βS9 phosphorylation levels in HT29 cells treated with ibuprofen and found them clearly increased, again in contrast to treatment with aspirin (Figure 4A). To confirm that the inhibitory phosphorylation of GSK3βS9 by ibuprofen is required for RAC1B downregulation, we used a non-phosphorylatable mutant of GSK3β [22]. This mutant has an alanine residue replacing serine 9, which generates a constitutively active GSK3β variant - GSK3βS9A. As shown in Figure 4B, the transfection of GSK3βS9A into HT29 cells clearly countered the effect of ibuprofen on RAC1B levels, confirming that GSK3β inhibition by S9 phosphorylation was involved in the ibuprofen-mediated decrease of RAC1B. Note that S9 phosphorylation of endogenous GSK3β still occurred in response to ibuprofen (p (S9) GSK3β in Figure 4B), but the overexpression of the constitutively active GSK3β mutant was sufficient to overcome that inhibition (Figure 4B). In order to confirm whether GSK3β activity was required for RAC1B generation, we inhibited endogenous GSK3β in cells using CHIR99021, the most selective, ATP-competitive inhibitor of GSK3 reported so far. As shown in Figure 4C, inhibitor treatment successfully abolished the autophosphorylation at Y216 and this inhibition of GSK3β activity was sufficient to cause a decrease in RAC1B protein to levels similar to those observed with ibuprofen treatment, but involving the alternative inhibitory S9 phosphorylation.

Moreover, confocal microscopy confirmed that the expression of GSK3βS9A also reverted SRPK1 (Figure 4D) and SRSF1 (Figure 4E) nuclear translocation despite the presence of ibuprofen. These data support a direct link between ibuprofen’s inhibition of GSK3β by S9 phosphorylation and the exit of SRPK1/SRSF1 from the nucleus, leading to a decrease in RAC1B splicing. Notably, the depletion by siRNAs of endogenous SRPK1 can inhibit RAC1b levels but did not affect the phosphorylation of GSK3β S9 (see Figure 6A), confirming that SRPK1 acts downstream of GSK3β.

**Figure 5:**
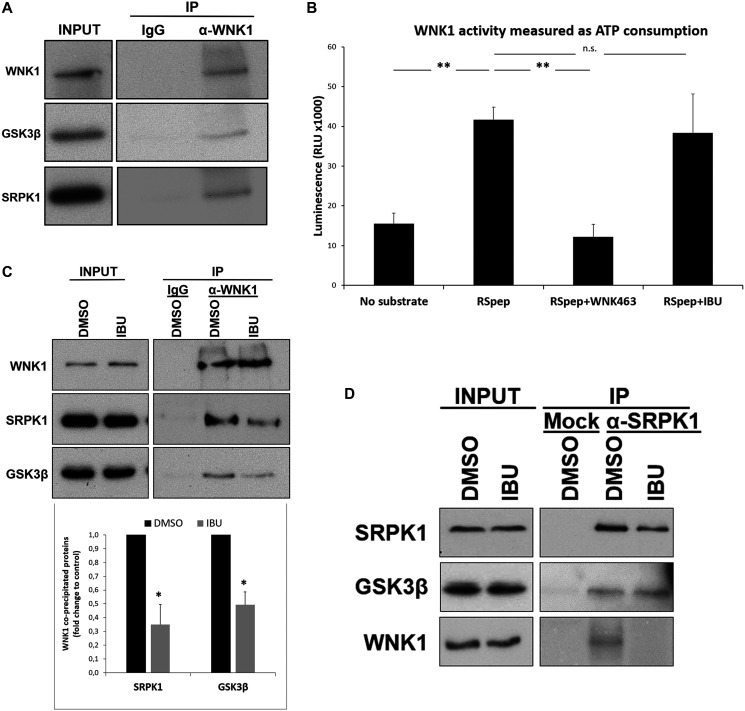
Ibuprofen impairs the interaction of WNK1 with GSK3β and SRPK1. HT29 cells were incubated with crosslinkers DSP and SPDP for 2 h in order to reversibly bind the network of proteins interacting with each other through short (DSP) or long (SPDP) spacers. After crosslinking, WNK1 was immunoprecipitated with anti-WNK1 antibody previously crosslinked to agarose beads with PFA 4%. Precipitated proteins complexes were de-crosslinked with 100 mM DTT, separated on a 9% (w/v) SDS-PAGE gel and transferred to PVDF membranes followed by western blot with the indicated antibodies. (**A**) Western blot analysis of proteins co-immunoprecipitating with WNK1. Note that GSK3β and SRPK1 co-immunoprecipitated with WNK1 suggesting an interaction between these proteins in colorectal cancer HT29 cells. (**B**) The ADP-Glo™ Kinase Assay kit (Promega) was used to assay the influence of ibuprofen treatment on the catalytic activity of WNK1, as described in the experimental procedures. A known WNK1 synthetic substrate RS Repeat peptide (Abcam) and the specific WNK inhibitor WNK463 (Carbosynth) were used as controls. Shown is a graphical display of the luminescence measured, representing the ATP consumption of the kinase reaction, from three independent experiments. Note that there was no significant difference between the ATP consumption for the kinase reaction with ibuprofen (RSpep+IBU bar) when compared to the control with DMSO (RSpep bar), but a significant decrease in the presence of specific inhibitor WNK463 (RSpep+WNK463 bar). All shown data represent means ± SEM.: ^**^ *P* < 0.01. (**C**) HT29 cells were incubated for 48 h with either DMSO control solvent or 500 μM ibuprofen, and then WNK1 was immunoprecipitated as described above. Shown is a Western blot analysis of proteins co-immunoprecipitating with WNK1 (upper panel) and a graphical display (lower panel) of the corresponding percentage of GSK3β and SRPK1 co-immunoprecipitated with WNK1 in three independent experiments. Note that GSK3β and SRPK1 co-immunoprecipitated with WNK1 in the control condition and that their amount decreased when IBU was present, suggesting that IBU disrupts the complex WNK1-GSK3β/SPRK1. All shown data represent means ± SEM.: ^*^ *P* < 0.05. (**D**) HT29 cells were incubated as described in (C) and then SRPK1 was immunoprecipitated as indicated above for WNK1. Shown is a Western blot analysis of GSK3β and WNK1 co-immunoprecipitated with SRPK1. Note that IBU treatment does not affect the amount of GSK3β that co-immunoprecipitated with SRPK1 suggesting that IBU acts through WNK1 (Ctrl: Without antibody; DMSO: dimethyl sulfoxide; IBU: ibuprofen; INPUT: whole cell lysate; IP: immunoprecipitation; RLU: relative light units; RSpep: RS repeat synthetic peptide; WNK463: WNK inhibitor).

**Figure 6:**
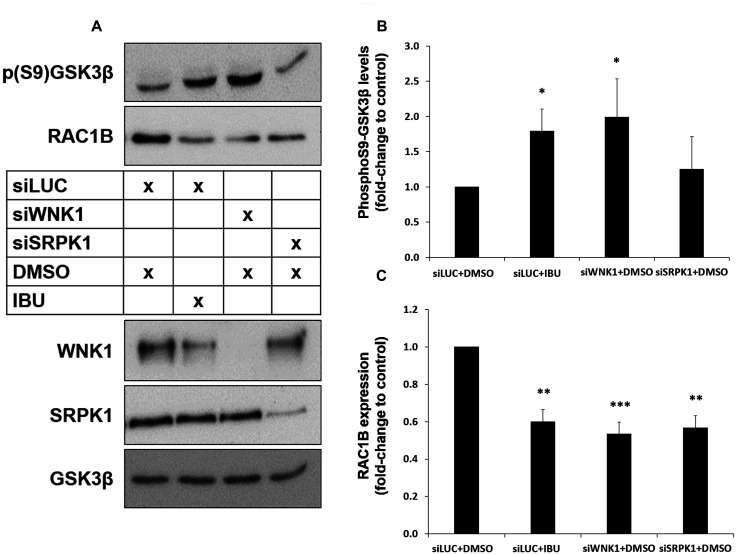
Both ibuprofen treatment or WNK1 depletion decrease RAC1B through GSK3β inhibition by S9 phosphorylation. HT29 cells were transfected with a control siLUC, siWNK1 or siSRPK1 and then incubated with DMSO (control solvent) or ibuprofen (500 μM) for 48 h. (**A**) Western blot analysis of whole cell lysates for the indicated endogenous proteins. (**B** and **C**) Graphical displays of the Western blot analysis of p (S9) GSK3β (B) and RAC1B (C) protein band intensities, quantified and normalized to siLUC+DMSO. All shown data represent means ± SEM, ^*^ *P* < 0.05, ^**^ *P* < 0.01, ^***^ *P* < 0.001 (DMSO: dimethyl sulfoxide; IBU: ibuprofen).

Together, these data identified GSK3β as upstream kinase involved in the regulation of alternative spliced RAC1B through SRPK1 phosphorylation and subcellular distribution, the latter of which then affect SRSF1 localization.

### Ibuprofen-mediated GSK3β inhibition involves impairment of WNK1-GSK3β/SRPK1 interaction

The mechanism of how ibuprofen affects RAC1B splicing did apparently not involve the direct inhibition of GSK3β’s catalytic activity, but rather another upstream event or partner. Intriguingly Sato and Shibuya reported recently that GSK3β can interact with and function as an effector downstream of the serine-threonine kinase WNK1 [23]. Therefore, we validated the interaction between WNK1 and GSK3β in HT29 cells by co-immunoprecipitation (Figure 5A). Interestingly, we found that beside GSK3β, SRPK1 also co-immunoprecipitated with WNK1. Because WNK1 could be regulating GSK3β or SRPK1 by phosphorylation in this complex, we performed again *in vitro* protein kinase assays to test if ibuprofen could be acting as an inhibitor of WNK1 activity. In contrast to the WNK-specific inhibitor WNK463, the presence of ibuprofen had no significant effect on WNK1 activity *in vitro* (Figure 5B). These results are in agreement with the observation by Sato and Shibuya that the stimulatory effect of WNK1 on GSK3β was not dependent on its catalytic activity but rather on their physical interaction [23]. In order to better characterize the protein complex, HT29 cells were treated with ibuprofen and WNK1 was immunoprecipitated. Consistently, we observed a significant reduction in the amount of both GSK3β and SRPK1 co-precipitating with WNK1 in HT29 cells treated with ibuprofen although total WNK1 protein levels in cell lysates were not reduced (Figure 5C). In contrast, ibuprofen treatment did not affect the interaction between GSK3β and SRPK1 when an anti-SRPK1 antibody was used for the immunoprecipitation (Figure 5D).

**Figure 7:**
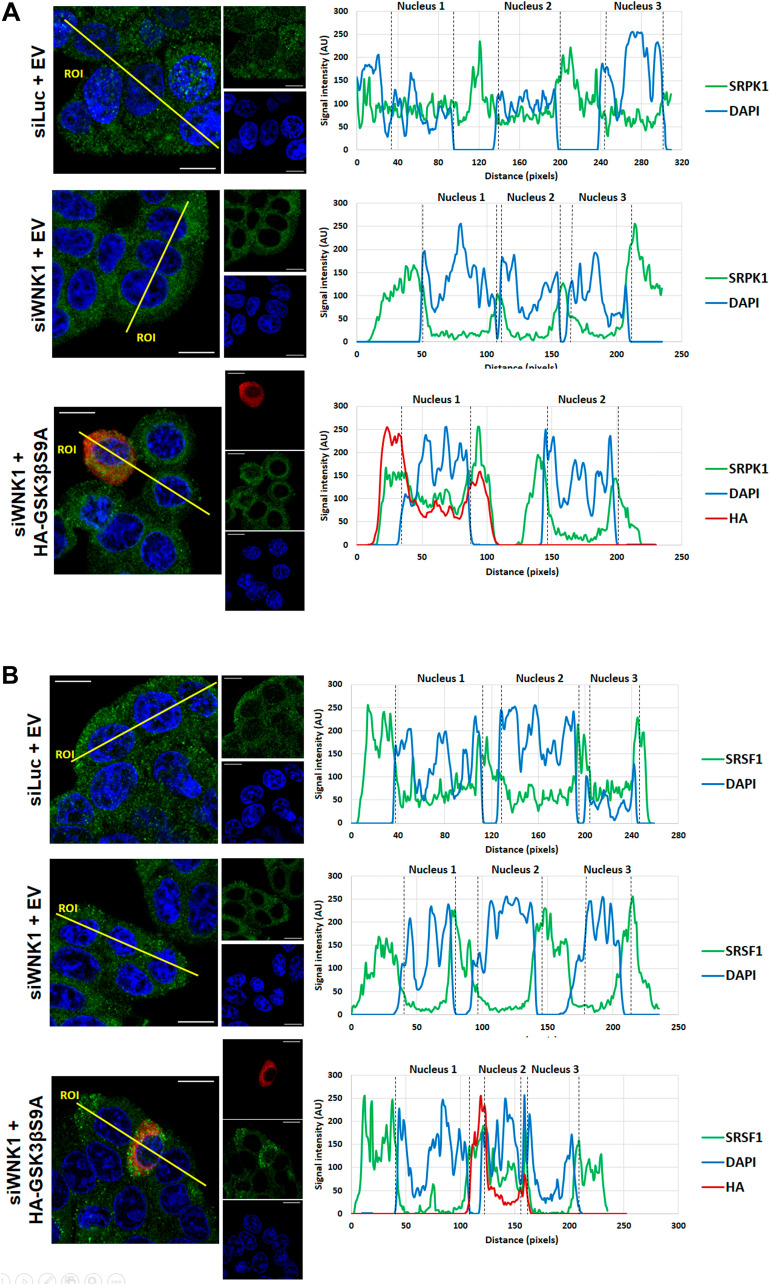
Effect of WNK1 depletion on SRPK1 and SRSF1 subcellular localization. HT29 cells were transfected with siLUC (control) or siWNK1, and 24 h later transfected with HA-tagged GSK3βS9A or a control empty vector as indicated. 48 h after the initial transfection, cells were analyzed by confocal immunofluorescence microscopy. Shown are the colored overlay and corresponding three confocal immunofluorescence images (left), which detected cell nuclei in blue (DAPI), the localization of (**A**) endogenous SRPK1 protein in green (anti-SRPK1 from BD Transduction Laboratories), or (**B**) endogenous SRSF1 protein in green (anti-SF2/ASF from Santa Cruz Biotechnology), and the transfected HA-tagged GSK3βS9A in red (anti-HA from Sigma-Aldrich). The nucleus and cytoplasm distribution of the three fluorescent signals was analyzed along optical sections (yellow lines) across several cells by plotting pixel intensities along the traced path (right graphs). In control and transfected HA-GSK3βS9A cells, SRPK1 and SRSF1 signals (green) were localized to both the cytosol and the cell nucleus (blue); however, in siWNK1-transfected cells, nuclear signals for SRPK1 and SRSF1 are nearly absent (AU: arbitrary units; DAPI: 4′,6-diamidino-2-phenylindole; DMSO: dimethyl sulfoxide; EV: empty vector; IBU: ibuprofen; SRPK1: serine/threonine-protein kinase; SRSF1: serine/arginine-rich splicing factor 1).

These data suggested that ibuprofen acts by removing WNK1 from a core GSK3β/SRPK1 complex, and this would facilitate the observed inhibitory phosphorylation of GSK3β at Ser 9. In order to provide evidence for this hypothesis, we downregulated endogenous WNK1 protein levels using a specific siRNA and wondered whether this could mimic the effect of ibuprofen. Indeed, depletion of WNK1 induced an increase in GSK3βS9 phosphorylation comparable to that produced by ibuprofen treatment (Figure 6A and 6B). Moreover, WNK1 knockdown was sufficient to decrease RAC1B expression levels to the same extent as ibuprofen treatment or SRPK1 knockdown (Figure 6A and 6C). Finally, we confirmed that the knockdown of WNK1 also reproduced the effect of ibuprofen on the subcellular localization of SRPK1 and SRSF1 (Figure 7A and 7B, respectively). Importantly, in WNK1-depleted cells, the simultaneous expression of GSK3βS9A rescued the nuclear localization of SRPK1 and SRSF1. These demonstrate that, as with ibuprofen, the effect of WNK1 depletion on inhibiting the SRPK1/SRSF1/RAC1B pathway depends on the increased GSK3βS9 phosphorylation (Figure 6A–6C).

**Figure 8:**
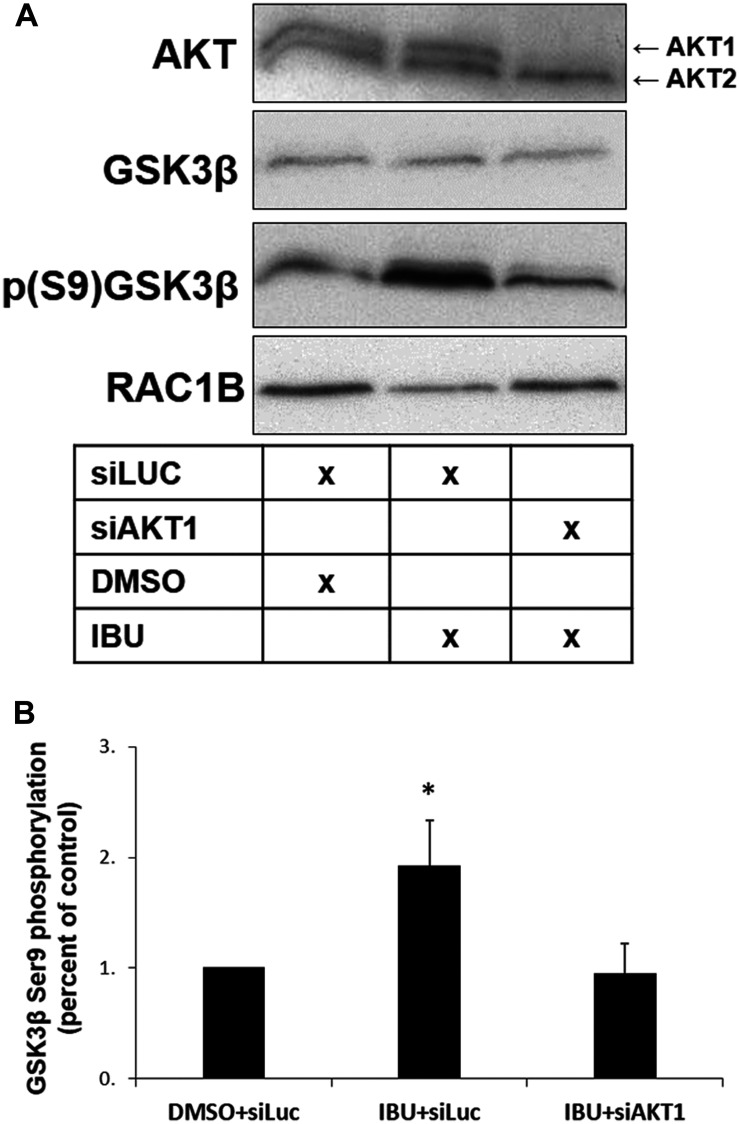
AKT1 is required for GSK3β S9 phosphorylation in ibuprofen-treated cells. HT29 cells were transfected with a control siLUC and treated with either DMSO (control) or ibuprofen (500 μM), or with siAKT1 and treated with ibuprofen (500 μM), all for 48 h. (**A**) Western blot analysis of whole cell lysates for the indicated endogenous proteins. (**B**) Graphical display of the Western blot analysis of p (S9) GSK3β protein band intensities after quantification and normalization to DMSO+siLUC. Note the reversion of the ibuprofen-mediated increase in GSK3β S9 phosphorylation after depleting AKT1. Cell transfection with siAKT1 alone but without subsequent IBU treatment had no significant effect on RAC1B levels [17]. All shown data represent means ± SEM, ^*^ *P* < 0.05 (DMSO: dimethyl sulfoxide; IBU: ibuprofen).

Together, these results indicate that ibuprofen treatment of HT29 cells impaired complex formation between WNK1 and GSK3β/SRPK1, which then allows GSK3β to become phosphorylated on its inhibitory S9 residue. In other words, GSK3β activity is apparently required to maintain translocation of both SRPK1 and SRSF1 to the nucleus, and this sustains RAC1B splicing. AKT1 is one of the protein kinases known to phosphorylate GSK3β on its S9 residue [24] so that we tested its involvement in our cell model. Upon siRNA-mediated depletion of AKT1, the ibuprofen-induced GSK3β S9 phosphorylation could not occur (Figure 8), suggesting that AKT1 activity is involved as soon as the complex WNK1-GSK3β/SRPK1 dissociates.

## DISCUSSION

Ibuprofen is one of the most widely used nonsteroidal anti-inflammatory drug (NSAID) and its antitumor properties have been largely attributed to inhibition of cyclooxygenase (COX) activities. Our results provide for the first time molecular insights into a COX-independent anti-tumor effect through which ibuprofen appears to affect alternative splicing of RAC1B. Our previous data had already described a specific inhibitory effect on RAC1B overexpression and suggested its action would be directly on the alternative splicing event [13]. Here we identified a molecular complex of three protein kinases, which controls the phosphorylation and nuclear translocation of splicing factor SRSF1 and is targeted by ibuprofen.

RAC1B alternative splicing is regulated by a splice enhancer element in exon 3b, which is directly bound by the splicing factor SRSF1, and an adjacent silencer element recognized by SRSF3 [25]. In human CRC cells, the availability of SRSF1 in the nucleus is the main factor regulating inclusion or skipping of exon 3b [17] and is modulated by its phosphorylation status. The protein kinase mostly responsible for SRSF1 phosphorylation is SRPK1, which is found both in the cytoplasm and in the cell nucleus [26, 27].

Previously, we described that pharmacological inhibition of SRPK1, or depletion of the endogenous SRPK1 protein by RNAi, decreased translocation of SRSF1 to the nucleus [17]. Here, we confirmed that ibuprofen also regulates RAC1B splicing by leading to decreased phosphorylation and translocation of SRSF1 to the nucleus (Figure 1A and 1C, respectively). However, ibuprofen treatment did not inhibit SRPK1 kinase activity directly, but rather interfered with its subcellular localization by depleting the nuclear SRPK1 pool (Figure 2C). This is compatible with a previous report showing that SRPK1 activity towards SRSF1 requires its own shuttling to the nucleus [20], where it may be required for nuclear retention of SRSF1.

Our previous analysis had also revealed that depletion of endogenous GSK3β inhibited the splicing of RAC1B [17]. We now provide evidence that GSK3β acts upstream of SRPK1. First, ibuprofen treatment of HT29 cells caused the inhibitory GSK3β S9 phosphorylation (Figure 4A), which determined the nuclear exclusion of SRPK1 and SRSF1 (Figure 4D and 4E). Second, we found that GSK3β can directly phosphorylate SRPK1 in an *in vitro* kinase assay (Figure 3), adding SRPK1 to the list of GSK3 substrates implicated in the regulation of alternative splicing, as recently reviewed by Liu and Klein [28]. The direct phosphorylation of SRPK1 may also explain why SRSF1 has not been reported as a direct target of GSK3β, in contrast with several other SR proteins like SRSF2 [29], SRSF9 and SRSF10 (TRA2β) [30]. Concerning the mechanism of ibuprofen action, a direct inhibitory action on GSK3β activity could not be detected (Figure 3). In contrast, we observed that ibuprofen disrupts the assembly of a protein kinase complex, composed of WNK1, GSK3β and SRPK1 (Figure 5C). In this complex, the physical presence of WNK1 was required; however, ibuprofen did not inhibit WNK1 kinase activity *in vitro* (Figure 5B). This is in agreement with the previous observation that the stimulatory effect on GSK3β was not dependent on WNK1 catalytic activity [23] and suggests it has a scaffolding protein role in assembling the GSK3β/SRPK1 complex. We provide strong evidence that WNK1 interaction with GSK3β protects the latter from inhibitory phosphorylation at S9. Thus, in the presence of WNK1, GSK3β remains active, allowing phosphorylation of kinase SRPK1 with subsequent nuclear translocation of SRPK1 and splicing factor SRSF1, until ibuprofen, or a yet uncharacterized physiological signal, disrupts the complex and triggers the observed changes leading to reduced RAC1B generation.

These results identify a previously unknown function of WNK1 in regulating alternative splicing-related proteins. WNK1 belongs to the WNK subfamily of protein kinases, which exists in multi-cellular organisms [31]. WNK1 was initially shown to regulate the activity of a variety of renal ion channels because *WNK1* gene mutations cause a rare familial form of hypertension [32, 33]. Subsequently, a variety of functions linking WNK1 to cancer cell biology have been identified and previously reviewed [34, 35], including activation of extracellular signal-regulated kinase 5 [36], modulation of TGF-β/Smad2 signaling [37], inhibition of autophagy [38], and increasing plasma membrane translocation of GLUT1 and cellular glucose uptake by phosphorylation of the RAB-GAP protein TBC1D4 [39]. Our data indicate a further role for WNK1 in the regulation of alternative splicing of tumor-related RAC1B, through complex formation with GSK3β and SRPK1. More detailed studies are required to determine whether ibuprofen binds directly to WNK1, leading to complex disruption, or whether other partners upstream of WNK1 are its molecular target. Following complex disruption, we found that the ibuprofen-induced GSK3β S9 phosphorylation could be mediated by AKT1, because it did not occur in AKT1-depleted cells (Figure 8). AKT1 is one of the protein kinases known to phosphorylate GSK3β on its S9 residue [24]. Consistently, we reported previously that interfering with AKT activity by overexpression of a kinase-dead AKT mutant increased RAC1B expression within 24 h [25]. Interestingly, the role of AKT in the regulation of the proposed protein complex might be more multifaceted: AKT has been reported to phosphorylate SRPK1 in its RS-domain [40], or to interact with and stimulate SRPK1 autophosphorylation [20]. In addition, AKT can phosphorylate WNK1 on threonine 60 [41, 42], although the physiological implications of this modification remain to be determined.

**Figure 9:**
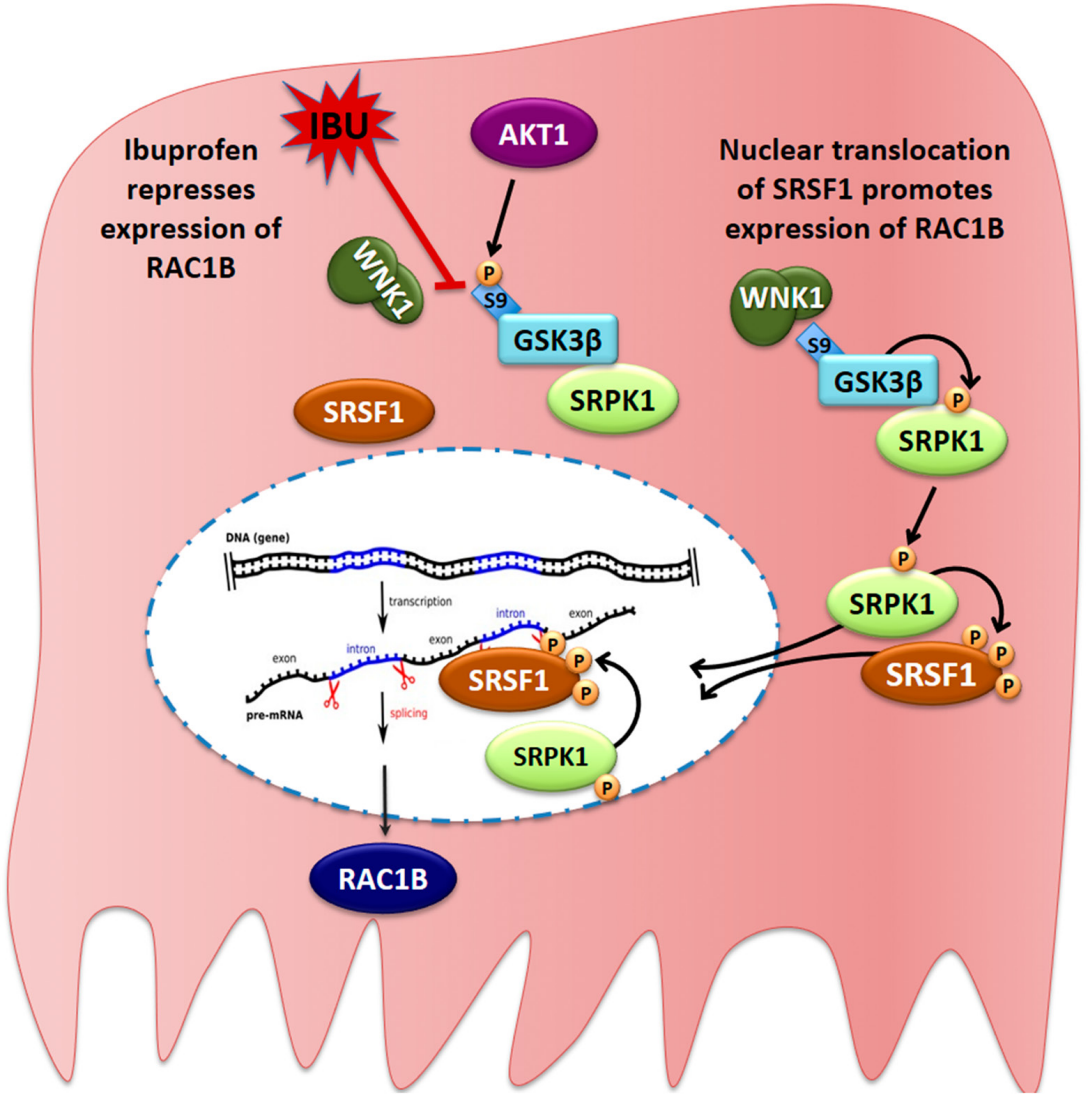
Proposed model for the ibuprofen-inhibited expression of alternative spliced RAC1B. HT29 cells represent a subgroup of colorectal cancer cells, in which alternative splicing of the RAC1 pre-mRNA yields a second transcript that becomes translated into the tumor-promoting RAC1B protein variant. In these cells, a cytoplasmic protein complex composed of WNK1, GSK3β and SRPK1 assures that GSK3β can phosphorylate SRPK1, allowing its translocation into the nucleus. SRPK1 phosphorylates the splicing factor SRSF1, first in the cytoplasm to promote its nuclear translocation, and then inside the nucleus to retain SRSF1 so that it can bind to its specific recognition motif in exon 3b of the RAC1 pre-mRNA. This promotes exon 3b inclusion and subsequent expression of alternative spliced RAC1B. In the presence of ibuprofen or after experimental depletion of endogenous WNK1, the interaction between WNK1 and GSK3β is disrupted, leading to exposure of the N-terminal serine 9 of GSK3β, which is then phosphorylated by AKT1. This phosphorylation is inhibitory and prevents GSK3β from phosphorylating SRPK1. As a result, SRPK1 remains cytosolic, nuclear phosphorylation of SRSF1 is no longer sustained, and SRSF1 leaves the nucleus so that exon 3b is no longer recognized by the spliceosome.

In conclusion, our data suggest that ibuprofen treatment interferes with a signal transduction pathway involved in the regulation of alternative spliced RAC1B. The proposed model is schematically depicted in Figure 9. One other report on prostate cancer cells receiving combined treatment of ibuprofen and epigallocatechin-3-gallate, reported changes in alternative splicing, in particular promoting the shorter and proapoptotic BCL-X (S) or MCL-1(S) variants [43].

The described effect of ibuprofen on alternative splicing of RAC1B involves the general splicing-regulatory proteins SRPK1 and SRSF1 and is most likely just the tip of an iceberg. It is known that splicing factors such as SRSF1 affect splicing of many genes [44, 45], but also RNA stability and translation, or post-translational sumoylation of other splicing factors [46, 47], so that the ibuprofen-induced changes in nuclear SRSF1 prevalence will most certainly affect a network of alternative splicing events [45, 48]. A more systematic genome-wide study should clarify the impact of ibuprofen treatment.

## MATERIALS AND METHODS

### Cell culture and transfection

HT29 cells were maintained in RPMI, supplemented with 10% (v/v) fetal bovine serum (FBS) (Invitrogen), and regularly checked for absence of mycoplasm infection. Cells were grown in 24-well plates or 35-mm dishes to 60-80% confluence, transfected using LipofectAMINE 2000 (Invitrogen), according to the manufacturer’s instructions, and analyzed 20–24 h later. Total amounts of transfected DNA were 0.5 μg per well of 24 well plates, or 2.5 μg of DNA for 35-mm dishes. If required, the amount of DNA was adjusted with empty vector. Plasmid transfection efficiencies were judged microscopically by expression of GFP and reached 40-60%. For RNA interference experiments, HT29 cells at 30%–40% confluence were transfected in 24-well plates or 35-mm dishes with 50 pmol or 250 pmol, respectively, of the indicated siRNAs using LipofectAMINE 2000 and analyzed after 48 h. SiRNA oligos were ordered from MWG Biotech with the following sequence: control siLUC: 5′-CGUACGCGGAAUACUUCGATT; siSRPK1: 5′-UUAUUCAGCAAGUGUUACATT; siWNK1: 5′-GCAGGAGUGUCUAGUUAUA. siRNA oligo for AKT1 was ordered from Santa Cruz Biotechnology (catalog number: sc-29195). For GSK3β inhibition HT29 cells at approximately 60% confluence were treated with 10 μM of CHIR99021 (BioVision, Inc; California, USA) during 48 h, using DMSO as control. HT29 cells at approximately 60% confluence were treated with 500 μM of Ibuprofen or 500 μM of Aspirin during 48 h (both from Sigma-Aldrich, Madrid, Spain), using DMSO as control.

### DNA plasmids

HA-GSK3β-S9A pcDNA3 was a gift from Jim Woodgett (Addgene plasmid # 14754) [22] and confirmed by automated DNA sequencing.

### Analysis of transcript expression

Total RNA was extracted from cell lysates with the RNAeasy kit (Qiagen, Hilden, Germany) and 1 μg reverse transcribed using random primers (Invitrogen) and Ready-to-Go You-Prime Beads (GE Healthcare). Real-time PCR quantification of alternative spliced RAC1B was based on amplification of two amplicons, one specific for RAC1B (78 bp; 5′-GGG CAA AGA CAA GCC GAT TG and 5′-CGG ACA TTT TCA AAT GAT GCA GG) and one for total RAC1 (75 bp; 5′-CCT GCA TCA TTT GAA AAT GTC CG and 5′- GAT GAT GGG AGT GTT GGG ACA GT as total RAC1 detector, i.e., RAC1+RAC1B) and each amplification performed in duplicate reactions and repeated in at least 3 independent experiments [25]. No amplification was obtained when RNA was mock reverse transcribed without adding reverse transcriptase.

### SDS-PAGE and western blotting

Samples were prepared and detected as described [49]. The antibodies used for Western blots were: mouse anti-α-tubulin (clone B-5-1-2), mouse anti-SR proteins (clone 1H4), and rabbit anti-HA from Sigma-Aldrich; rabbit anti-RAC1B from Millipore; mouse anti-PCNA from MerckBiosciences; mouse anti-SRPK1 and mouse anti-β-catenin from BD Transduction Laboratories; rabbit anti-AKT, rabbit anti-GSK3β (27C10) and rabbit anti-PhosphoGSK3β (Ser9) (D85E12) from Cell Signalling; mouse anti-p-GSK-3α/β (6D3) (sc-81496), mouse anti-SF2/ASF (96) (sc-33652) (SRSF1) and anti-Histone H2b (sc-10808) from Santa Cruz Biotechnology and sheep anti-WNK1 from Dundee University. For the detection of phospho-SRSF1, total SRSF1 was first immunoprecipitated with the specific anti-SRSF1 antibody (sc-33652) and this enriched fraction then probed with the-SR phosphoepitope antibody (1H4). For densitometric analysis of band intensities, X-ray films from at least three independent experiments were digitalized and analyzed using ImageJ software (NIH).

### Cell fractionation

Nuclear proteins were separated from cytosolic proteins using a cellular fractionation protocol that we will describe next. Briefly, cells were scraped and lysed on ice for 5 min in 300 μl of cytosol buffer [50 mM HEPES at pH 7.2, 2 mM EDTA, 10 mM NaCl, 250 mM Sucrose, 2 mM DTT, 0.05% (v/v) NP40 and a protease inhibitor cocktail (Sigma)]. The cytosolic fraction was collected by centrifuging the lysate at 6000 rpm for 4 min (at 4°C) and adding the supernatant to 60 μl of 5× Laemmli SDS sample buffer. The pellet containing the nuclear fraction was washed once in cytosol buffer and then resuspended in 75 μl of nuclear buffer [50 mM HEPES at pH 7.2, 2 mM EDTA, 400 mM NaCl, 20% (v/v) Glicerol, 2 mM DTT and a protease inhibitor cocktail (Sigma)]. The resuspended nuclear fraction was incubated on ice during 20 min (with occasional vortexing) and collected by centrifuging the lysate at 10000 rpm for 10 min (at 4°C) and adding the supernatant to 15 μl of 5× Laemmli SDS sample buffer. Equal volumes of both fractions were analyzed side by side on Western blots. Results were confirmed in at least three independent experiments.

### Immunoprecipitation

HT29 cells were seeded in 100 mm culture dishes at near 100% confluence, placed on ice and washed three times with ice cold PBS-CM (PBS pH 8.0 containing 1 mM CaCl_2_ and 1 mM MgCl_2_). Cells were then incubated with the crosslink solution DSP+SPSP (1:1 0,2 mM; Thermo Scientific) for 2 h. To quench the reaction 50 mM Tris/HCl pH 8.0 was added for 15 min. Cells were again washed 3× with cold PBS-CM and lysed in 750 μL lysis buffer (50 mM Tris/HCl pH 7.5, 2 mM MgCl_2_, 100 mM NaCl, 10% (v/v) glycerol, 1% (v/v) NP40) supplemented with a protease inhibitor cocktail composed of 1 mM PMSF, 1 mM 1,10-phenanthroline, 1 mM EGTA, 10 μM E64, and 10 μg/mL of each aprotinin, leupeptin, and pepstatin A (all from Sigma-Aldrich). Cell lysates were cleared at 3000× g for 5 min at 4°C. The supernatant was pre-cleared by adding 45 μL streptavidin-agarose beads (Sigma-Aldrich), rotated for 1 h at 4°C and centrifuged for 1 min at 3000× g. Approximately 700 μL of the pre-cleared lysates were recovered and added to the beads prepared as described in the following section (see Beads preparation) and rotated overnight at 4°C. After incubation, the tubes were centrifuged for 1 min at 3000× g and washed 6× in cold wash buffer [50 mM Tris/HCl pH 7.5, 2 mM MgCl_2_, 150 mM NaCl, 10% (v/v) glycerol, 1% (v/v) NP40]. Captured proteins were recovered in 50 μL 2× SDS modified sample buffer with extra DTT (100 mM) and analyzed by SDS-PAGE and Western blotting as described in the corresponding section.

### Beads preparation

60 μl of dry G-protein agarose beads (Roche) were incubated with 1 μg of mouse anti-SRPK1 (BD Transduction Laboratories) or 1.5 μg of sheep anti-WNK1 antibody (Dundee University) diluted in PBS for 1 h at 4°C. Beads were washed 3× with PBS and crosslinked to the antibody with 4% (v/v) formaldehyde in PBS for 1 h at 4°C. After crosslink, beads were washed 3× with PBS and blocked with 2% BSA in PBS solution rotating for 1 h at 4°C, washed 3× with PBS and added to the pre-cleared lysate recovered as described in the Immunoprecipitation section.

### Confocal immunofluorescence microscopy

Cells were grown on 10 × 10 mm glass coverslips to about 20–30% confluency, incubated and transfected as indicated above, then washed twice in PBS, immediately fixed with 4% (v/v) formaldehyde in PBS for 60 min at room temperature, and subsequently permeabilized with 0.5% (v/v) Triton X-100 in PBS for 30 min at room temperature. Cells were then labeled for 60 min with either a 1:500 dilution of SRPK1 antibody (BD Transduction Laboratories) or a 1:100 dilution of SRSF1 antibody (anti-SF2/ASF (96) (sc-33652) from Santa Cruz Biotechnology), combined with a 1:250 dilution of HA antibody (Sigma-Aldrich) for HA-GSK3βS9A transfected cells, washed 3 times in PBS for 5 minutes with gentle shaking, followed by 30 minutes incubation with a 1:250 dilution of Alexa Fluor 488 and rabbit Alexa Fluor 546 (both from Invitrogen). Coverslips were washed 3 times in PBS, briefly stained with 0.5 ng/ml DAPI (Sigma), washed again and then mounted in VectaShield (Vector Laboratories) and sealed with nail polish. Images were recorded with the 405 nm, 488 nm and 546 nm laser lines of a Leica TCS-SPE confocal microscope and processed with Adobe Photoshop software.

### ADP-Glo™ kinase assay

This assay is more sensitive for measuring low to moderate protein kinase activities as it amplifies in an enzymatic conversion step the levels of ADP that result from the phospho-transfer. ADP-Glo™ Kinase Assay kit was purchased from Promega. The primary reaction consisted of the kinase, substrate and ATP in 60 mM HEPES, pH 7.5, 3 mM MgCl_2_, 3 mM MnCl_2_, 3 μM Na_2_VO_4_, 1.2 mM DTT and 0.05 mg/ml BSA. After 1 h of incubation at 30°C, 10 μL of ADP-Glo reagent was added and incubated for 40 min at room temperature. Then, 20 μL of kinase detection reagent was added and, after an incubation time of 30 min, luminescence was measured on a GloMax^®^ 96 microplate luminometer (Promega). All the kinases used were purchased from ProQinase. For WNK1 20 ng of kinase, 3.25 μg of RS Repeat peptide (Abcam) and 100 μM ATP were used, for SRPK1 50 ng of kinase, 1.5 μg of RS Repeat peptide (Abcam) and 2 μM ATP. For each kinase reaction, two control reactions were made: one without substrate and other without kinase. 500 μM ibuprofen (Sigma-Aldrich) was added to the reactions, using DMSO as control. Specific inhibitors for each kinase were 10 μM WNK463 (Carbosynth) for WNK1, and 25 μM SRPIN340 (kindly provided by Dr. Masatoshi Hagiwara) for SRPK1.

### Kinase-Glo^®^ luminescent kinase assay

This assay is more accurate for measuring higher protein kinase activities as it directly measures the consumption of co-factor ATP during the phospho-transfer. Kinase-Glo^®^ Luminescent Kinase Assay kit was purchased from Promega. The primary reaction consisted of the kinase, substrate and ATP in 60 mM HEPES, pH 7.5, 3 mM MgCl_2_, 3 mM MnCl_2_, 3 μM Na_2_VO_4_, 1.2 mM DTT and 0.05 mg/ml BSA. After 1 h of incubation at 30°C, 20 μL of Kinase-Glo^®^ Reagent was added and mixed at room temperature for 10 min, and luminescence was measured on a GloMax^®^ 96 microplate luminometer (Promega). All the kinases used were purchased from ProQinase. For GSK3β we used 140 ng of kinase, 1 μg of GSM (Millipore), or 1 μg of SRPK1, and 1 μM ATP. For each kinase reaction, control reactions were made without substrate or without kinase. 500 μM ibuprofen (Sigma-Aldrich) was added to the reactions, using DMSO as control. As specific inhibitor for GSK3β we used 10 μM CHIR99021 (BioVision). Note that all the above conditions included also 25 μM SRPIN340 (kindly provided by Dr. Masatoshi Hagiwara) in order to inhibit SRPK1 kinase activity.

### Statistical analysis

Data were analyzed using Student’s *t*-tests for paired samples or ANOVA tests followed by post-hoc Tukey’s tests when comparing multiple treatments. *P* < 0.05 was accepted as the level of statistical significance. Shown data reflect the mean ± SEM from at least three independent experiments.
